# Inclusivity in Insomnia: Adolescents’ Perspectives on the Sleep Solved App: Qualitative Interview Study

**DOI:** 10.2196/82410

**Published:** 2026-06-10

**Authors:** Sarah E Bennett, Grace M Lewis, Stephanie Chambers, Milly H Johnston, James Denison-Day, Anthony Duffy, Georgia Treneman-Evans, Paula Kuberka, Nicholas Christoforou, Lee M Ritterband, Robert Meadows, Doaa Alamoudi, Ian Nabney, Lucy Yardley

**Affiliations:** 1 Department of Psychology, University of Bath, Bath, England, United Kingdom; 2 School of Psychological Science, University of Bristol, 12a Priory Road, Clifton, Bristol, England, BS8 1TU, United Kingdom, +44 117 428 248; 3 Health Protection Research Unit, Bristol Medical School, University of Bristol, Bristol, England, United Kingdom; 4 Centre for Academic Child Health, Faculty of Health and Life Sciences, Bristol Medical School, University of Bristol, Bristol, United Kingdom; 5 School of Psychology, University of Southampton, Southampton, England, United Kingdom; 6 School of Interactive Arts and Technology, Digital Health Circle Lab, Simon Fraser University, British Columbia, BC, Canada; 7 Department of Psychiatry and Neurobehavioral Sciences, Center for Behavioral Health and Technology, University of Virginia Health System, Charlottesville, VA, United States; 8 Department of Sociology, University of Surrey, Guildford, England, United Kingdom; 9 Department of Management Science, Yanbu Industrial College, Yanbu, Saudi Arabia; 10 School of Engineering Mathematics and Technology, University of Bristol, Bristol, England, United Kingdom

**Keywords:** behavior change, digital intervention, insomnia, depression, anxiety, sleep, qualitative research, adolescents, underserved populations, co-created digital app, sleep hygiene, mobile phone

## Abstract

**Background:**

Adolescent sleep duration can substantially impact mood, behavior, and academic attainment. While hundreds of sleep-related apps are available to download, none have been cocreated with adolescents from underserved populations in the United Kingdom.

**Objective:**

This study aimed to explore adolescents’ views, expectations, and experiences with a novel app to improve sleep, called Sleep Solved, to understand which features were perceived as positive and helpful, and to identify ways to further enhance its usefulness. Sleep Solved is part of a larger stepped behavior change study and was cocreated with adolescents from underserved groups to make the app accessible and engaging for this population.

**Methods:**

A total of 63 participants aged 16‐18 years from across the United Kingdom completed semistructured interviews after trying the app. Interviews were analyzed using inductive thematic analysis, as outlined by Braun and Clarke, with a particular focus on the views of individuals from underserved ethnic and socioeconomic groups.

**Results:**

Participants perceived Sleep Solved as a useful tool that provides helpful advice regarding changeable behaviors to improve sleep hygiene. Cocreated features of the app, such as the Sleep Stars gamified rewards system and the easy-read, science-based “sleep hacks,” were viewed positively by participants, who reported that they had a beneficial impact on their sleep and sleep schedule. Praise was given for the app’s ease of use and how the science of sleep was explained at an appropriate level, without being overwhelming. Compared to sleep advice on social media platforms, Sleep Solved was considered more reliable and trustworthy. Participants described better sleep hygiene, such as a regular sleep routine and a longer sleep duration, and increased feelings of improved mood and energy.

**Conclusions:**

This study found that a cocreated sleep app, designed with input from adolescents in underserved UK populations, was perceived as accessible, reliable, and effective in supporting positive sleep behavior change. Although sleep duration was not objectively tested, participants, particularly those from low socioeconomic status backgrounds and diverse ethnicities, reported improved sleep routines and mood, highlighting the potential of co-designed digital tools to engage and benefit adolescent users.

## Introduction

Adolescents in the United Kingdom report significant insomnia symptoms, with prevalence rates from 4% [1] to 23.8% [2]. This is a major public health concern as sleep difficulties can lead to poor academic results [3,4], reduced school attendance [5], and impacts on mood and social and family life [6].

Since the “perfect storm” of biological, psychological, and sociocultural factors in adolescent sleep biology was first described by Carskadon [7], the field of adolescent sleep biology has evolved at a rapid pace [1].

Environmental factors such as social pressures, examinations, and anxiety surrounding academic performance can affect adolescents’ sleep [2,3]. Thanks to the near ubiquity of mobile phone use among adolescents, socializing can now occur around the clock, into the early hours of the morning. Adolescents who are more “popular,” those receiving the largest number of phone notifications, have also been found to have a shorter sleep duration, and for girls, more reported symptoms of insomnia [4]. Light-emitting technologies have also been flagged for concern, for the effects that these can have on sleep, and the wider influence of technology on sleep [5]. Social pressure, poor social skills, and a lack of parental supervision may drive problematic social media use, where adolescents can face peer pressure to stay up late and engage with friends [6,8-10].

Person-centered methodologies, or “coapproaches” such as coproduction, co-design, and cocreation [11], are methods used to bring people together as active and equal partners in research [12]. A principal element in the person-based approach (PBA), cocreation involves academics working collaboratively to produce interventions that are relevant and acceptable to stakeholders, perceived as useful, and are therefore more likely to be adopted in “real world” settings [13]. In this study, the cocreation of the Sleep Solved app by and for adolescents in the United Kingdom, using the PBA [14], has been published previously [15,16]. Our prior paper detailed how the app was successfully cocreated and optimized in partnership with adolescents to coproduce an app that was accessible, representative, and easily understood, but without requiring extensive engagement for users to benefit from a range of sleep hygiene advice and support. In addition to the PBA, the behavior-change intervention is underpinned by 2 key theories: Bandura’s Social Cognitive Theory [17], to support the behavioral elements of Sleep Solved and aim to improve users' sleep-related self-efficacy, and the socioecological model of sleep health, to represent the multifactorial nature of a variety of moderators on sleep, at the individual, social, and societal levels [15,16,18].

For example, sociodemographic moderators can include socioeconomic status (SES), which has been strongly associated with poorer sleep outcomes in adolescents. In a systematic review, adolescents from low SES families, those on lower incomes, or with more indicators of poverty, were more likely to develop sleep disorders, including difficulty initiating or maintaining sleep, and were significantly more likely to develop insomnia [19]. Factors such as lower neighborhood safety and greater worries about community violence have been linked to a bigger risk of developing sleep problems in adolescence [20-23], whereas neighborhood contentedness, living in a safer environment, or a more affluent area, has been associated with a longer sleep duration [21,24,25]. Within the home, low SES adolescents may also face difficulties with family overcrowding, a lack of structure or routine, or high levels of sensory stimuli, such as noise, factors which can also lead to poorer sleep quality and increased sleepiness [26,27].

Building upon these socioeconomic influences, research has also shown potential sleep differences across different ethnic groups, which can further complicate adolescent sleep patterns. White participants have had better sleep outcomes compared to minority groups. Black, Asian, and Hispanic participants tended to have shorter sleep durations and problems with waking and sleep onset [28]. In a similar study of sleep duration, Black and African American participants reported an average of 15.1 minutes less sleep compared to White participants [18,29].

Sociodemographic influences on sleep can also intersect. Research has indicated that those belonging to a minority ethnic group and a socioeconomically deprived household may be more likely to experience poor sleep, and thereby poorer health outcomes [18,30]. Being of a minority ethnic group and living in a low SES household has been associated with shorter self-reported sleep duration [31] and greater reported sleep complaints [32]. Inequalities in physical and environmental factors, such as the built environment, pollution, noise, walkability, access to healthy food, and social factors such as crime, safety, and social connectivity, have all been shown to affect sleep in both adult and pediatric populations [23]. Contextual factors, such as ethnicity and SES, will be important elements to consider when examining the effectiveness of any new sleep intervention.

A key factor in the success of any intervention is participant engagement. Having a low SES has been linked to reduced participant engagement with app-based interventions [13], such as mHealth (mobile health) apps that target healthy eating or physical activity [33,34]. Adolescents with low SES are also at higher risk of poorer physical health behaviors, such as low levels of physical activity, smoking, high-energy food intake, and drug misuse, compared to children from families with a higher SES [35-38]. They may have lower English language skills compared to children with a higher SES [39]. In addition, research has indicated that young people, immigrants, those with a basic education, on a low income, and receiving government benefits are significantly more likely to have poor health literacy and potentially worse health outcomes [40]. Therefore, to mitigate any potential user difficulties with lower literacy levels, lower health literacy, and reduced participant engagement, attention to the needs of those from lower SES regions was considered a vital factor in the design and co-creation of Sleep Solved. The app was carefully refined throughout to be at an easy-read level, at an approximate reading age of 9‐10 years (Flesch-Kincaid score of 90‐100, aged 10‐11 years, or a fifth grade level, as measured by the Readability Scoring System version 2.0) [41]. Difficult concepts such as hormone changes or circadian rhythms were explained through simple language and pictures, to reflect different learning styles and improve engagement. Based on patient and public involvement feedback and behavior change theory, a social modeling element was created, where audio clips from users with different regional accents explained how the Sleep Solved app had worked for them [15,16].

Research has indicated a bidirectional link between sleep difficulties in adolescence and mental health problems such as anxiety and depression [17]. Adolescents with low SES have been shown to have higher rates of depressive symptoms and medication use and were more likely to receive a mental health diagnosis through adolescence and into adulthood [42]. The highest prevalence of mental health diagnoses has been found in adolescent boys with low SES, whereas adolescent girls of medium to high SES have been shown to have the lowest prevalence [43]. Sleep disturbances have also been associated with later suicidality risk among adolescents. At the age of 11 years, children who reported sleeping fewer than 8 hours a night had a higher risk of suicidal thoughts and suicide attempts at the age of 18 years, when compared to adolescents who slept for 9 hours or more [44]. With embarrassment and discomfort potentially acting as a barrier to seeking formal mental health support [18-20], offering accessible sleep hygiene advice to adolescents through mHealth apps and their own smartphones may be a less stigmatizing way to provide support. This is particularly true as an estimated 99% of adolescents in the United Kingdom have access to the internet, and 90% of those aged older than 11 years own a smartphone [21].

To create relevant and applicable interventions for adolescents that are also engaging, it is vital to involve the perspectives and lived experiences of underserved communities in research, including those from low-income or minority ethnic families [45,46]. To ensure that research interventions have the best chance of being effective, a wide variety of different people need to be recruited to take part in research, especially from groups that are not involved in research as often [45,47]. This also ensures that any findings from the research could be more generalizable to a broader population. While many apps that promise to aid sleep are available to be downloaded, few have been cocreated with input from adolescents themselves [48,49]. Cocreating with the people who will be using them is intended to ensure that the app is relevant, meets their needs, is accessible and engaging, and therefore will be more likely to be used successfully [50].

The purpose of Sleep Solved was to serve as an accessible first step of a stepped program of support. Adolescents shown to have more persistent sleep difficulties, anxiousness, or low mood could then be guided toward the extensive support provided by existing apps: SHUTi (Sleep Healthy Using the Internet) [51], Bite Back [52], and Project YES (Youth Empowerment and Support) [52]. As with Sleep Solved, there are some notable examples of apps in existing research, which have been cocreated with young people to improve their sleep, such as the SMILE (Smile for Life) app [48] and Sleep Ninja for adolescents with insomnia [49]. The only example from the United Kingdom is Sleep SSI (single session intervention), a one-off intervention cocreated with 11 adolescents, based on the Project YES app [53,54]. However, none of these sleep apps have been cocreated with adolescents from underserved ethnic groups or from areas of social disadvantage. In addition, only small groups have provided feedback on their user experience. In order to address these limitations, there is a need for all users to recognize people such as themselves and their social and cultural experiences, to engage with, and feel included in an accessible app [55,56]. Sleep Solved addresses these limitations within the literature through the engagement and involvement of a wider range of underserved UK adolescents in app development, and the use of coproduction methods in order to ensure the user voice in app development and design.

The purpose of this paper is to understand users’ experiences of the Sleep Solved app while taking part in a large feasibility trial. It was important to explore how the app was perceived, how users engaged with the content, determine which cocreated features of the app were enjoyed and considered helpful, and identify ways in which its usefulness might be further improved.

## Methods

### Study Design

This paper explores adolescents’ (aged 16‐18 years) perspectives of a new, cocreated stepped digital intervention to support sleep hygiene behaviors and well-being. In-depth qualitative interviews were conducted, focusing particularly on a diverse range of adolescents from across the United Kingdom.

### Ethical Considerations

Ethics approval for this study was granted by the North-West–Greater Manchester Central Research Ethics Committee (23/NW/0129). All methods were completed in accordance with applicable guidelines and regulations. Participants were informed about this study’s aims and objectives, procedures, their right to withdraw from this study, and how their data would be stored in line with the General Data Protection Regulation in this study’s information sheet. All participants provided verbal informed consent before taking part in a one-to-one qualitative interview. All data were anonymized, and all identifying information was removed. Only the research team had access to deanonymized data, which was stored on secure password-protected servers in accordance with General Data Protection Regulation requirements. In compensation for their time, interview participants were offered a digital voucher of £40 (US $53.93), which was financed through this study’s budget.

### Participant Recruitment

Adolescents aged 16‐18 years were recruited to a nonrandomized feasibility trial carried out in collaboration with 33 schools and colleges from across the United Kingdom. Participants were invited to participate if they were aged between 16 and 18 years and owned a smartphone. Those receiving treatment for insomnia or a diagnosed mental health condition were excluded from this study, as they were receiving treatment. Participating schools and colleges received a “welcome pack” on enrolling in this study, featuring a study poster, a digital presentation explaining this study, staff and student information videos, and a document outlining the most effective ways to recruit students. Participants could express their interest in taking part by scanning a QR code or by following a direct link on the poster, presentation, or videos to the participant information sheet and consent form.

On initial sign-up to this study, and at 6 and 12 weeks, participants completed several quantitative measures to assess their levels of insomnia, anxiety, and depression; the Insomnia Severity Index [57-59], and the Hospital Anxiety and Depression Scale [60] or Revised Child Anxiety and Depression Scale [61], for users aged younger than 16 years. These findings are explored in our later papers in this series. Details of the recruitment of participants to the nonrandomized feasibility trial can be found in Multimedia Appendix 1. The focus of this paper is on the qualitative interviews conducted as part of this study, namely participants’ feedback on their use of the cocreated Sleep Solved app.

For the interviews, purposive sampling was used to better reach underserved populations in research. All those who had expressed an interest in being interviewed were purposively sampled based on ethnicity and levels of socioeconomic deprivation. The levels of deprivation were estimated by comparing contributors’ postcodes to the 2019 English indices of deprivation, a relative measure of deprivation for small areas (lower-layer super output areas [LSOAs]) [15,62,63]. To ensure that adolescents from ethnically diverse and disadvantaged backgrounds were well represented, involvement of participants from diverse ethnicities and more socioeconomically disadvantaged areas was prioritized for participant interviews.

### Data Collection

Interview participants provided written consent to take part in a one-on-one interview using televideo software. Participants were allocated a unique identification number, and the data were anonymized, whereby all identifying information was removed. Participants were informed about the purpose of this study, the benefits and possible downsides of participating, the apps, what would be required of them, how their data would be stored, and who was funding the research, and had the opportunity to ask questions. Talking with the research team about this study in a one-to-one interview was entirely optional, and only those who consented to be contacted about an interview and for the interview to be recorded were invited to participate. Participants were reminded that they could stop participating at any time, without giving a reason. Names and corresponding identification numbers and consent forms were kept separately and securely on a password-protected server.

A semistructured interview topic guide was used to guide discussions, developed by members of the research team (SEB, MHJ, GT-E, PK, LY, and RM). Open-ended questions explored participants’ reasons for participating, sleep experiences before and after trying the apps offered, as well as their knowledge of sleep hygiene advice and any experiences of sleep advice. Participants were asked about app use, app content, use of social support, and whether they were likely or not to continue to use the app. To ensure relevance of the questions to adolescents, they were further refined after the first 10 interviews for clarity and to capture any new factors. A relationship with participants was not established before the commencement of this study.

Interviews were carried out by televideo between November 2023 and April 2025 by 5 women members of the research team: SEB, GML, PK, SC, and MHJ. All had prior training in and experience of conducting in-depth qualitative interviews. As the team conducting the qualitative interviews was all women, this may have influenced both data collection and analysis. Being of the same shared gender as many of our participants might have influenced rapport, particularly when discussing behaviors around sleep experiences. In line with the approach by Braun and Clarke [64] to inductive thematic analysis, possible biases were considered throughout data collection and analysis, through ongoing group discussion and reflection. Interviews were conducted on televideo, enabling participants to see the characteristics of the interviewer. Interviews were recorded using televideo software, transcribed verbatim, anonymized, and checked for correctness. Each participant was assigned a participant information number. Data collection concluded at 63 interviews, where no additional factors were identified from the data, and a rich awareness of the issues raised had been achieved [65].

### Data Analysis

Qualitative analysis was conducted iteratively by 3 women members of the research team (SEB, GML, and SC) using a pragmatic inductive thematic analysis approach, a method to identify, analyze, and report patterns within qualitative data [64]. NVivo (version 14; Lumivero), a qualitative data analysis management software, was used to manage the data [65]. All 3 coders had prior training in thematic analysis, coding, advanced use of NVivo software [65], and qualitative research methods. The codes, which were inductively derived from the data by each researcher, rather than any preexisting framework, were combined to form themes, and this process continued until a series of overarching themes or key findings were recognized. Preliminary results and a thematic map were shared and discussed with the wider research team to further refine the themes identified [64]. Any disagreements were discussed with a fourth member of the research team, who has substantial coding and qualitative research experience, in order to achieve thematic validity. Data saturation was considered to have been realized when no further aspects were identified from the data [65]. This was achieved at 55 interviews and continued to 63 participant interviews to ensure that no new ideas were introduced. These results have been reported in line with COREQ (Consolidated Criteria for Reporting Qualitative Research) reporting guidelines (see Checklist 1) [66].

## Results

A total of 63 participants took part in semistructured interviews; self-identified gender indicated 44 (69.84%) women, 17 (26.9%) men (one of whom identified as female to male transgender), 1 (1.58%) “prefer not to say,” and 1 (1.58%) “no response.” The mean age of the participants was 16.90 (range 16‐18, SD 0.75) years. Table 1 gives an overview of age, self-identified gender, ethnicity, and indices of deprivation data in this sample (N=63).

**Table 1.** Age, self-identified gender, ethnicity, and indices of deprivation data in this sample (N=63).

Demographic characteristic	Interviewed participants
Age (years)	
n (%)	
16	21 (33.33)
17	27 (42.85)
18	15 (23.80)
Mean (SD)	16.90 (0.755)
Gender, n (%)	
Young man	17 (26.98)
Young woman	44 (69.84)
Transgender	1 (1.58)
Prefer not to say	1 (1.58)
No response	1 (1.58)
Ethnicity, n (%)	
White English, Welsh, Scottish, Northern Irish, or British	34 (53.96)
Asian or Asian British	18 (28.57)
Bangladeshi	4 (6.34)
Indian	3 (4.76)
Pakistani	8 (12.69)
Black, Black British, Caribbean, or Black African	3 (4.76)
Black African	2 (3.17)
Black Caribbean	1 (1.58)
Mixed or multiple ethnic groups	4 (6.34)
White and Asian	2 (3.17)
White and Black Caribbean	1 (1.58)
Any other mixed or multiple ethnic background	1 (1.58)
Other ethnic group: Arab	2 (3.17)
Indices of Deprivation (2019 English Indices of Deprivation data), n (%)	
10% least deprived	2 (3.17)
20% least deprived	3 (4.76)
30% least deprived	5 (7.93)
40% least deprived	4 (6.34)
50% least deprived	4 (6.34)
50% most deprived	9 (14.28)
40% most deprived	2 (3.17)
30% most deprived	10 (15.87)
20% most deprived	13 (20.63)
10% most deprived	11 (17.46)
Total most deprived	45 (71.42)
Total least deprived	18 (28.57)

Table 2 compares the self-defined ethnicity of those interviewed for this study (N=63) to the high-level ethnic groups in England and Wales recorded in the 2021 UK Census [67], indicating a good relative ethnic heterogeneity of participants across those interviewed, compared to national data.

The mean duration of the interviews was 40.34 (SD 10.579; minimum 22, maximum 67) minutes. The levels of relative regional deprivation were estimated using contributors’ postcodes against the 2019 English Indices of Deprivation Data [62,63]. The Index of Multiple Deprivation (IMD) considers 7 subsections of deprivation: income, employment, education, health, crime, access to housing and services, and living environment [62]. The Indices of Deprivation are a measure of deprivation at small local level areas (LSOAs). These LSOAs are a standard statistical geography designed to contain a similar population size; approximately 650 households, equating to around 1500 residents [68]. Most of the participants interviewed for this study (45/63, 71.42%) lived in the 50% most deprived regions of the United Kingdom. Levels of relative deprivation in our sample varied from 10% of the most deprived neighborhoods (11/63, 17.46%) to 10% of the least deprived (2/63, 3.17%).

**Table 2.** Self-defined ethnicity in this sample (N=63), compared to the 2021 UK census data.

Self-defined ethnicity	Interviewed participants, n/N (%)	2021 UK census data: high-level ethnic groups in England and Wales [67] (million), n (%)
White English, Welsh, Scottish, Northern Irish, or British	34/63 (53.96)	44.4 (74.4)
Asian or Asian British	18/63 (28.57)	5.5 (9.3)
Black, Black British, African, or Caribbean	3/63 (4.76)	2.4 (4)
Mixed or multiple ethnic groups	3/63 (4.76)	1.7 (2.9)
Other ethnic group	1/63 (1.58)	1.3 (2.1)

Three overarching themes are presented as follows: (1) reasons for participating and expectations of the Sleep Solved app, (2) experiences of Sleep Solved, and (3) perceived benefits of Sleep Solved. A quantified proportional summary of themes, split by number of participants, number of codes, SES, and ethnicity can be found in Multimedia Appendix 2.

### Reasons for Participating and Expectations of the Sleep Solved App

#### Poor Sleep

Most of those interviewed described having a poor sleep schedule, such as an erratic sleep pattern, as their reason for taking part. Many cited that they had “nothing to lose” in participating, and that they were willing to “give it a try” to find out if Sleep Solved made any difference to their sleep:

*Well, my sleep can’t get any worse than it already is, so I might as well try it.*

[018, Black, Black British, Caribbean or African: African, young woman, aged 16 years, 20% most deprived]

Some participants described being told about the Sleep Solved app by friends and classmates, who were taking part in this study too, whereas others were recommended to the app by perceived authority figures, such as college teaching or well-being staff.

*I talked to like a member of* [Wellbeing] *staff before because I was having a lot of trouble with sleeping and she mentioned it to me…what made me kind of want to use it because she kind of explained everything about it and asked if I wanted to do it. And I thought it would be quite interesting…I have like a regular meeting with her and she mentioned it during one of them.*

[006, White, young woman, aged 17 years, 40% least deprived]

*I was referred to the Wellbeing team at my college and I spoke to a woman who mentioned the Sleep study and said that I’d probably be beneficial…As in my case would be helpful…How I can sleep better? How I can stop stressing about sleeping? Like how I can stop worrying about my sleep?... The woman who recommended* [Sleep Solved] *to me said that there’s a few of the people that tried it and it helped them…it’ll give me more of a peace of mind to the fact that I’m not alone in it, and there’s more people around the world and even near me, like at college that struggle with it as well.*

[038, White, young woman, aged 18 years, 30% least deprived]

#### Incentives and Interests in Research

The multiretailer voucher offered to participants was described as “a good incentive” to take part, as was the fact that this study seemed both “interesting” and trustworthy, as it was based at a university:

*The fact that like it’s a study for university and I think that’s to me that sounds really fun.*

[009, White, Woman, aged 18 years, 50% most deprived]

*I'm a psychology student in college, so it just sounded like an interesting opportunity.*

[018, Black, Black British, Caribbean or African: African, young womanoman, aged 16 years, 20% most deprived]

#### Unsure of What to Expect

Of those interviewed, the majority were unaware of what Sleep Solved could do for them or the potential benefits. However, some anticipated that the Sleep Solved app would provide them with helpful advice and tips on how to improve their sleep and their sleep hygiene:

*I just I was expecting like an app which it was and be like tips to help me which it was! So yeah, it did meet all my expectations.*

[003, White, young woman, aged 18 years, 30% least deprived]

*I just wanted... tips and how could get to sleep faster and how I could also have, quality sleep and not just quantity.*

[019, Asian or Asian British: Indian, young man, aged 16 years, 20% most deprived]

#### How Sleep Solved Could Help Them

Participants suggested that improved sleep could be achieved through developing a better sleep routine and getting sustained, better-quality sleep. Others described more negative preconceptions that engaging with the app would be a long, drawn-out activity, or that, as this study was “science-based,” they would be forced to talk with large groups of people.

*I think I was expecting this long lengthy process where I have to talk with loads of people on a meeting, and be like a science experiment or something! (laughs)… It was a lot easier than I thought it would be...I thought that I’d have to talk to quite a few people, but it’s a lot easier than I thought.*

[014, White, young woman, aged 18 years, 20% most deprived]

However, many participants were pleasantly surprised at how easy the app was to use, and the ways that their sleep schedule changed as a result of using Sleep Solved:

*I wasn’t expecting anything to be perfectly honest with you. I was like when it says something about sleep, I thought like it’s just going to, like improved it for a little bit? I wasn’t expecting any like, huge results or anything, but once I got into the study itself, and I actually did what like the app was telling me to do. I was like, surprised by ...how that impacts my sleeping schedule...I was surprised. Yeah, I was amazed!*

[013, Asian British (Other), young woman, aged 18 years, 40% most deprived]

### Experiences of Sleep Solved

#### Accessibility and Usability of Sleep Solved

Participants described the app as quick, accessible, enjoyable, and easy to navigate. They found that the sleep-based science presented and explained in the app was pitched at an appropriate level and was easy to understand, compared to other apps they had tried: “Just the right amount of information...[to] benefit from it, without getting overwhelmed” [038, White British, young woman, aged 18 years, 30% least deprived]. Although some participants had feared that the science-based nature of the app might be overwhelming or complicated, many were pleased to find the explanations provided to be simple and easy to understand.

*Perfect the way it is, and it’s very simplified. Like, it doesn’t go into a long, detailed explanation like a whole paragraph... Easy to understand, wish I … found this sooner. I think it’s really simplified. It’s just really good and it’s helped me massively.*

[034, White British, young woman, aged 16 years, 30% most deprived]

*I thought that would be like a biology lesson, to be honest with you, always frightening. Well... it actually did make sense. And like with the graph, all this kind of stuff, because I thought “Oh, it’s a biology lesson. No thank you!” I would never be able to understand it, but that was very brave. Like, I understood everything.*

[013, Asian or Asian British: Any other Asian background, young woman, aged 18 years, 40% most deprived]

Of our sample, around a quarter of those interviewed had no experience of the sleep advice presented in Sleep Solved before using the app, and often found the advice to be novel, “interesting,” and presented in ways they had not encountered before.

*I did feel more confident. And like with all the information I learned a few things. Which is nice. I don’t think- if I hadn’t joined Sleep Solved, I don’t think I ever would have got around to doing that. (laughs)*

[018, Black, Black British, Caribbean or African: African, young woman, aged 16 years, 20% most deprived]

#### Credible Source of Information

The sleep hygiene advice presented in Sleep Solved was perceived as more trustworthy and “reliable,” compared to other sources of information, such as social media.

*I felt like it would like I could really connect to it. I could relate to it because, it was like, the tips and then everything was like kind of tailored to like people my age. I think that was so much better than what I’ve seen in like social media and stuff*

[058, Asian or Asian British: Indian, woman, aged 18 years, 50% least deprived]

*When there’s … a 14-year-old on TikTok telling you they’re doing this before you go to sleep is going to help. Whereas if it’s like a trusted study that shows that actually getting 8 hours of sleep and not using your phone or blue light or anything actually does improve your sleep, it’s a lot more easy to identify* [as trustworthy]

[039, mixed ethnic group: White and Black Caribbean, young woman, aged 17 years, 40% least deprived]

Users found the app enjoyable to use and the content interesting and appealing.

*I thought it was quite interesting. Like I remember like clicking on them like the sleep advice like how to do better sleep...then like just being, like, intrigued by them. And it was just a bit quirky, like just different, you know, like just the quirkiness. And like you don’t actually see that often.*

[004, White, young woman, aged 17 years, 30% most deprived]

Participants in this intervention had the option to earn “Sleep Stars” when using the Sleep Solved app, an optional gamification element cocreated with adolescents. Users who tried collecting Sleep Stars during this study described them as a “fun” and appealing challenge and a good incentive for them to keep engaging with Sleep Solved.

*I’m such an easily entertained person. The fact that you can click* [a] *star and it turns blue is more than enough for me to do it every day. And so, yeah, I definitely think…It’s more of an incentive to do it, rather than just clicking a button that says, “Yes, I’ve used the hacks” and going on with my day.*

[032, White British, woman, aged 18 years, 30% most deprived]

Some in our sample explained taking advantage of social support, comparing their scores with friends, and setting themselves challenges to improve their sleep.

*I’ve got 2 gold stars, and I’m working to get my third. I quite like it, it feels like a challenge to sleep well…I quite like that. I’m a very competitive person, so I’m determined to beat all my friends, so I’ve got making sure I get more stars than they do! We’ve made it into a little bit of a challenge, almost.*

[012, White British, young woman, aged 17 years, 20% least deprived]

#### Reasons for Nonengagement

A small number of interviewed participants (9/63, 14.3%; 8 of Asian British ethnicity and 1 of Black British ethnicity) did not engage with Sleep Solved. Reasons for nonengagement included forgetting to use the app, so the inclusion of app notifications and reminders may mitigate some of these barriers to participation in future versions of Sleep Solved. For some, Sleep Solved was less relevant, as they already slept well, but they felt that the app could be useful for those with poor sleep.

*Nothing made me not want to use it…I think it was quite beneficial, but of course it’s beneficial for people who like need it, I guess.*

[037, Asian or Asian British: Pakistani, young woman, aged 17 years, 20% most deprived]

Table 3 provides an overview of the content provided in each of the sections of the Sleep Solved app, with selected participant quotes.

**Table 3.** Sleep Solved app content and representative quotations from participants.

Sleep Solved content	Description	Selected quotes
App content overall: science-based learning about sleep	Participants enjoyed the “science-based” information provided by Sleep Solved. They described the scientific elements presented as easy to read and understand, and accessible, compared to more difficult ideas they had encountered in the past.	“I mean, they pulled out a lot of science and that made sense, credit to them, they explain the kind of science elements very well. It wasn’t . really health jargony. It was very straightforward. and I, I got it.” [030, White British, young woman, aged 18 years, 30% most deprived] “Having the ‘why’ right next to the to the solution was nice.Because, like people would say, ‘Don’t use caffeine before bed’.And I’d say, ‘But why though?’ .So, then I wouldn’t listen because I’m like, ‘Well, you didn’t know what you’re talking about. So why should I listen?’ But with the app, it definitely sounded like the tips knew what they were saying, because there was the reason.” [013, Asian British (Other), young woman, aged 18 years, 40% most deprived]
Sleep hack: “How can less time in bed help me sleep better?”	The “spending less time in bed to sleep better” hack explained the “three kinds of sleep” (deep sleep, dreaming sleep, and light sleep) and recommended that participants spend less time in bed to sleep, using their bed for sleeping rather than for being on their phone, planning for the next day, or eating. Participants agreed that this made sense and that the hack had been an easy change to make. Needing 5‐6 hours of deep or dreaming sleep to sleep well also made sense as a target.	“At first I was like, ‘That feels a bit counter-intuitive’ and my skeptical brain kicked in, but then I remember like it was about like cortisol levels and like, training your brain that you bed is a place to sleep and not do other things, which I was like, ‘OK, that makes sense.’” [018, Black, Black British, Caribbean or African: African, young woman, aged 16 years, 20% most deprived] “Of all the hacks.that has been my godsend .not crawling into bed to sit on my phone. I’ll do anything. I’ve been sitting on my desk more and I think it’s made it a lot easier for me to actually fall asleep. That’s definitely my fave hack.” [012, White, young woman, aged 17 years, 20% least deprived]
Sleep hack: “Why could sleeping in make me feel bad?”	Cortisol: facts about sleep hormones, including the “get up and go” hormone “cortisol,” which helps you to feel alert, including how getting up at the same time every day trains the brain to release cortisol at the “right” time. Participants understood the links between changes in their sleep schedule and later feelings of hunger, overeating, or low mood to be related to cortisol. The page was described as easy to understand, with praise for the simple graph and easy-to-read science.	“It was a good explanation. It kind of made sense, cause when I wake up later, a lot of the time I feel worse. Yeah. So, it’s part of the reason why I’ve been trying to wake up more consistently earlier. I knew about the effects of it, but I didn’t know that was linked with cortisol.” [008, White, young woman, aged 18 years, 50% most deprived] “That’s quite a good visual representation of it. .with the graphs, like, it does kind of captivate you a bit and make you want to kind of look at it more.[a] visual aid can just make it more like interesting to read because you’ll see something. like with graphs and things and you just want to read what it’s about.” [028, White British, young woman, aged 17 years, 30% most deprived]
Sleep hack: “Why could sleeping in make me feel bad?”	Is it OK to nap when I’m tired? Participants were advised to only nap before 3 PM, keep the curtains open, and limit their napping to 20 minutes or less. Many had not heard this advice before, using this as an opportunity to either reduce the amount of time they were napping or to stop napping during the day entirely, due to their newfound awareness of the effect on their sleep later in the evening. For others, this advice was less relevant as they did not nap during the day.	“I thought it was actually really good because for me I sometimes accidentally take naps like I’m just sitting and I slowly start slipping away into the sleep. And I didn’t realize. So, when I go to bed later, I’m not able to go to sleep. I forgot to mention that cause I stopped napping altogether because I felt like it wasn’t helpful. when I did nap earlier in the day, I don’t even know what times I would nap, I just felt like no matter when I slept, I could never sleep better at night.” [018, Black, Black British, Caribbean or African: African, young woman, aged 16 years, 20% most deprived]
Sleep hack: “How can I stop worrying in bed?”	Getting up after 20 minutes if unable to sleep: Many described a range of activities they had tried when using this sleep hack, including getting warm drinks, reading books, or craft activities. For some, leaving their bed was too much of a challenge, whereas others were concerned about waking up family members or pets in the house. They described limiting activities to their bedroom, rather than going downstairs to avoid disturbing others.	“The advice did work quite a bit cause I used [to] like walk downstairs and sit on the sofa, spend time with my family, and that then went up to bed. It did work. I got to sleep a lot quicker than normal.” [020, White British, young man, aged 17 years, 10% most deprived] “Yeah, because I don’t want to wake up anyone else in the house. not if I’ve got on my phone. But if I start walking through the house and stuff. Get something to eat or drink. if I wake the dog then he’ll start being annoying to my parents.” [005, White, young woman, aged 16 years, 10% least deprived]
Training your brain to be calm: military sleep hack, relaxing music, and sounds	The “military sleep hack” encouraged progressive muscle relaxation, “tensing and then relaxing every muscle in your body really does help you fall asleep,” and relaxing music to get to sleep more easily.	“[The Military Sleep Hack] I’ve definitely been using at least four times a week now to go to sleep, if not more. I’m just able to relax and I just go to sleep after that, like within minutes, which is really nice. And I do realize how useful it was until I’ve tried it.it definitely relaxes my body really fast and really nicely.” [056, White: Any other White Background, young man, aged 17 years, 10% most deprived]
Challenges and suggestions for future versions	Users had some suggested ideas for how Sleep Solved could be updated in future versions.	“Notifications. Don’t think I’ve got any notifications. Like in evening, it’d be like ‘Ohh, it’s time to sleep.’” [027, Asian or Asian British: Indian, Woman, aged 18 years, 50% most deprived] “It’s not like the app itself is difficult, it’s more if I just forget if I’m like in a rush in the morning” [012, White, Woman, aged 17 years, 20% least deprived] “To be honest, I really have bad memory. I set out with this whole thing, and I was like, ‘OK, my sleep‘s bad, needs sorting out. Maybe downloading the app will help,’ and then I’ve just completely blanked and haven’t done it.” [014, White, Woman, aged 18 years, 20% most deprived]

Suggestions by participants to improve usability and engagement with Sleep Solved included widening the current age range (14‐18 years) to include older and younger participants, as people can experience sleep problems at any age.

*Doesn’t matter what your age is, you can suffer from poor sleep. So, like, a little toddler could suffer from poor sleep, old women could suffer from sleep? And I think, like, it should be promoted more because there is a lot of people in the world who do, like, suffer from sleep* [problems]. *So, if the app is made, the app could help them more than, like, usual tips I guess.*

[021, Asian or Asian British: Pakistani, young woman, aged 17 years, 10% most deprived]

A minority of those interviewed suggested improving the notifications and reminders that participants received to re-engage with the app, as forgetting to use and engage with Sleep Solved was listed as one of the most common reasons for not using the app while being enrolled in this study.

*I tried to do the one week challenge, but yeah I didn’t get any notifications from the app… you know, Duolingo, they’ve got like a fairly interactive user interface. And then the reminders are very like fun…I think that could motivate me to open… the app and do more stuff as well.*

[025, Asian or Asian British: Any other Asian background, young woman, aged 17 years, 30% most deprived]

Ideas for the gamification elements within the app included adding high scores and a leaderboard to add an element of competition.

*I know it may be very effective, like if I was in a competition with someone else? Oh my God! I would- I would be sleeping at 8:00 PM every single day! Yeah, I would do that…making it competitive, more competitive would be nice, because that’s how I play… I’m just so competitive and just get so stressed if I don’t win. So obviously if I was playing with the Sleep Solved study, I would be sleeping not at 8:00 PM at 6:00 PM!*

[013, Asian or Asian British: Any other Asian background, young woman, aged 18 years, 40% most deprived]

### Perceived Benefits of Sleep Solved

#### Effects on Mood and Behavior

Participants in this study, most of whom were from low SES groups and underserved populations, described feelings of improved mood and energy, potentially as a result of improving their sleep schedule, while others described feeling more relaxed.

*I didn’t* [think sleep was important] *until I started sleeping more. And now I do 100%. More happy, more energetic and just more, just more energetic really. Happier…A lot more happier.*

[034, White British, young man, aged 16 years, 40% most deprived]

*I kind of have like been in a higher mood. Than before I start* [Sleep Solved].

[051, Asian British, young woman, aged 16 years, 20% most deprived]

Some in our sample spoke about how they were now more productive in the mornings. Compared to their poor sleep before using Sleep Solved, they are now able to have a calmer start to their morning, setting them up for the day ahead with less stress, worry, and anxiety.

*I’ll make myself some breakfast ... for the rest of the day. I feel so much better. That I’ve had a drink and I’ve had something to eat. And I’ve like had time to get ready instead of rushing, which then stresses me out... I just feel a lot calmer.*

[037, Asian or Asian British: Pakistani, young woman, aged 17 years, 20% most deprived]

#### “Toolbox” to Help With Sleep

Sleep Solved was described as a useful “tool,” formula, or “recipe” to help with sleep, with most of these interviewees being from the 10%‐50% most deprived regions of the United Kingdom. To remind themselves of the sleep behaviors, users described checking back in with the app occasionally to prompt themselves, or during periods when they have difficulty sleeping. Others relied upon the sleep hacks to guide them during periods where they were not sleeping as well:

*I think I will, if I feel like I’ve forgotten how to help my sleep. At the moment I do think I’ve like, I’ve got it tracked, like I have the recipe for good sleep because I haven’t had a bad sleep in a while, … I have the recipe down for a good sleep. Obviously when I go to university … I probably use Sleep Solved again to help me figure out what routine would help.*

[018, Black, Black British, Caribbean or African: African, Woman, aged 16 years, 20% most deprived]

Since trying the Sleep Solved hacks, interview participants from the most deprived areas of the United Kingdom described achieving a more regular sleep routine, feeling less tired, waking at the same time every day, and having perceptions of deeper sleep. Many had dramatically improved the amount they were sleeping each night, from as little as 3 to 5 hours of sleep per night, to 7 to 9 hours of sleep each night, after using the Sleep Solved app. Most of these participants were from the most socioeconomically deprived areas of the United Kingdom.

*Using the Sleep Solved hacks. That has helped me…when I usually get up to about five hours of sleep I’m normally getting now say about 7 hours… I’ve been sleeping for a lot longer than normal…Because if I use* [Sleep Solved], *it’ll benefit my sleep and like, help me get better sleeping instead of like a couple of hours*

[020, White British, young man, aged 17 years, 10% most deprived]

For 1 participant with chronic back pain and arthritis, the change in his sleep had been particularly remarkable, with prescriptions for sleep medication no longer necessary since using Sleep Solved:

*There was a massive difference. I was sleeping for three hours before. And I slept for 9 hours last night. I would say* [I’m sleeping more than 9 hours] *mostly every night now. Now I’ve gone back to* [my doctor], *and said that there’s been a bigger change, and they no longer see* [sleep medication as] *necessary. He said it’s a massive increase, which could ultimately help my back* [pain] *with more rest.*

[035, White British, young man, aged 16 years, 30% most deprived]

#### Sharing Sleep Solved With Others

When asked, most of those interviewed were keen to continue to use Sleep Solved going forward, whereas others had shared the sleep hacks with friends and other members of their family who had difficulty sleeping.

*I’ve told my mum more, kind of about it than anyone else, but she thought* [Sleep Solved] *was like quite interesting and cool because I was showing her all of it. And then I kind of talk to her about it, like in the morning when I have to, like, pick what you want… she thinks it’s like, really good and kind of interesting because, I mean, she sometimes struggles sleep as well. So I was gonna, like, recommend us some of the hacks to her.*

[006, White British, young woman, aged 17 years, 40% least deprived]

*So I’ve mentioned it to four friends, five? and from that I think, two, three of my friends have decided to join the study now…. Yeah, it’s cause I’ve seen good effects from* [Sleep Solved]. *So I was like, why not? Whenever I see good effects from something or I really enjoy it, I usually recommend it to my friends. And because I’m like, pretty nice and trustworthy person, they listen to me… if they can’t get the app, or they just don’t want to for some reason, I’m able to advise from my perspective for what I’ve seen, it works, or I think might work for them nicely.*

[056, White: Any other White background, young man, aged 17 years, 10% most deprived]

## Discussion

### Principal Findings

This paper aimed to explore adolescents’ views, expectations, and experiences of Sleep Solved, with a particular focus on members of the underserved populations that it was intended to benefit. The positive experiences of and engagement with Sleep Solved described by this sample of adolescent participants were encouraging, and many reported substantial benefits for their mood and sleep. The methods recommended in the Sleep Solved sleep hacks, including regular sleep and wake times [69], getting up after 20 minutes if unable to sleep (thereby reducing the amount of time spent in bed) [70], progressive muscle relaxation [71,72], and mindfulness meditation [73], are all evidence-based sleep behavior change techniques that have been successfully used in adult populations. From these results, it is clear that Sleep Solved has the potential to be effective in adolescent, underserved, and low SES populations.

Low SES has been linked to significantly poorer user engagement with mHealth apps, including those that target health behaviors [13,33,34]. Most of those interviewed from low SES or underserved groups joined as they experienced poor or irregular sleep. As some had not heard of Sleep Solved or its potential benefits, they were motivated to take part by recommendations from close friends or college staff. The need for trusted authority figures to refer those who may need it to the Sleep Solved app is an important finding, as adolescents experiencing poor sleep or mental health may not always be aware of supportive interventions, or seek them out themselves [74,75]. People are substantially more likely to engage in a digital health intervention when it is recommended to them by a recognized authority figure, such as a trusted friend, health professional, or the government [76-78]. Considering this, and the need for underserved and low SES adolescents to be well supported into the intervention, future referral pathways for Sleep Solved would benefit from being promoted by an authority figure or trusted peer. Research has shown that participants who hold positive expectations about an mHealth intervention are more likely to find the intervention effective and engage more in using the app [79]. Therefore, the need for the potential benefits of Sleep Solved to be framed positively will be crucial for any future rollout.

Overall, the challenges faced by participants from low SES and underserved populations, such as the potential for low literacy levels, lower English language skills, poor reading comprehension, or low health literacy, were well met by the Sleep Solved app. Participants appreciated the accessible, science-based information provided by Sleep Solved, with praise for its readability and ease of understanding. Great care was taken to make Sleep Solved as accessible as possible to users from underserved groups, particularly those with lower literacy levels and lower health literacy [15,16]. Similar research with underserved groups has found that users with low literacy skills can struggle to understand some of the language used in online interventions, even text perceived as “simple” by the research team [80], highlighting the need for effective coproduction at all stages of the research project [15,16]. These efforts to make the app more inclusive, understandable, and easy to use have the added benefit of improving the usability of the app for all participants, not just those who may have difficulties with reading or understanding [80].

The “sleep hacks” provided by Sleep Solved were seen as being more legitimate and trustworthy than the sleep advice they had seen on social media platforms. How adolescents trust health information on social media can be complex [81,82], with many placing more belief in health-related information from legitimate websites, compared to social media or networking sites [82]. Participants in this study also spoke of learning about sleep and sleep-related advice on social media platforms. The quality of health-related information on social media apps used by adolescents in this study is low [83,84]. As promotional materials for the current study made clear that it was being conducted as university research, this may have influenced participants’ positive perceptions of the Sleep Solved app. The advice in Sleep Solved might have therefore been seen as more trustworthy [74], removing the need for users to continuously appraise and monitor the accuracy, relevance, and credibility of the information presented to them.

Gamification, the chance to collect Sleep Stars when using Sleep Solved, was also a popular motivating feature of the app, encouraging friendly competition between users. The results from Sleep Solved are similar to prior research in the area of sleep apps. For example, adolescents using the Sleep Ninja app can work toward a black belt in sleep at the end of a 6-week program [75], whereas users who meet their sleep and wake-up time goals in the Sleep Town app could collect a variety of buildings and coins [76]. Engaging in gamification can enhance feelings of reward and accomplishment when achieving a target behavior, such as keeping to a regular sleep schedule [76,77]. However, much of the existing research in this area is based on the experiences of adults, raising questions about the degree of applicability to adolescents [69]. Factors relating to adolescent sleep discussed previously, such as later circadian rhythms, social pressures, examination stress, and the readiness of phone use, may require more targeted adaptation to better meet the needs of adolescents. Gamification, including engaging in social comparison, rewards, and challenges, may be more engaging, motivating, and interesting for adolescents, but this can vary, and feedback from the adolescents who will be the target users of the app is essential [85-87].

Social relatedness is also an important factor in popular sleep hygiene game designs, such as the use of friending, social prodding, and group quests [88]. While social relatedness was not used directly as a game design feature in Sleep Solved, interviewed participants spoke of a degree of social competitiveness and requested a leaderboard and the ability to form friendship groups in future iterations of the app. In addition, adolescents have previously expressed a preference for gamified elements; self-tracking behaviors that reward progress toward their goals, increasing their motivation to engage with the app and regularly track their progress [78].

Changes to behavior and health, such as those promoted by our Sleep Solved cocreated sleep intervention, can potentially lead to “ripple effects”: outcomes relating to the intervention that were not an explicit goal of the intervention, but that can have a positive impact [89,90]. These positive ripple effects can extend beyond participants themselves to their familial and social domains [89-92]. In addition to their own use of Sleep Solved, participants spoke of sharing the app and the sleep advice contained within with others who had difficulties sleeping, such as family members or friends. This included social connections who would not have met the initial age range (14‐18 y) or inclusion criteria for this study. As numerous participants had observed helpful effects from Sleep Solved, they described sharing the app with others who they felt could benefit from getting better sleep and from following the sleep hacks recommended in the program. Successful social diffusion of mHealth apps has also been seen in other interventions, such as in apps for HIV protection [93]. These findings mirror participants’ recommendations to widen the availability of Sleep Solved, from being available to those aged 14‐18 years to older and younger participants. Other apps that address maladaptive sleep behaviors, such as SHUTi, have been successfully cocreated and culturally tailored to be inclusive and relevant to a range of demographic groups, including American adults [51], Black women [94], those with caring responsibilities [95], older adults [95], and adolescents in the United Kingdom [15].

### Limitations

Although this study took great care to purposively sample adolescents from a variety of ages, genders, ethnicities, and levels of socioeconomic deprivation, our findings will not be reflective of all UK adolescents’ experiences. Although we purposively sampled interviewees who had not engaged with the app, people who had more positive experiences and views of the app will have been more likely to agree to be interviewed. For this reason, while the encouraging views expressed by many of our interviewees provide an indication of the benefits it can provide, particularly for adolescents from underserved and low SES populations, these findings cannot be used to generalize or estimate the likely benefits at a population level. In addition, the reported benefits of the app are subjective; the duration of participants’ sleep was not objectively measured during this study, instead relying on self-reported changes in sleep behavior over time. Future work could mitigate the effects of these limitations. For example, a large randomized controlled trial would mitigate the effects of self-selection bias by randomly selecting participants to take part in the intervention, potentially engaging with users who had fewer positive experiences or who did not engage with the Sleep Solved app. Future mixed methods results, exploring both quantitative and qualitative data, would enable any patterns to be investigated in greater depth.

### Future Work

Many participants expressed a desire to continue using Sleep Solved in the future, even beyond the end of this research study. User enthusiasm for the Sleep Solved app, and participants reporting positive promotion of Sleep Solved to friends and family members, was an unexpected but encouraging finding. Future iterations of Sleep Solved could potentially be tailored to different age ranges, such as versions for children, young adults, or older adults.

### Conclusions

This study of Sleep Solved and wider Sleep Well intervention has shown promising results in a varied range of underserved and ethnically diverse adolescents with sleep difficulties from across the United Kingdom. Sleep Solved was perceived as easy to use, with interesting and appealing content that was easy to read and understand. The adaptation of the app for users with low literacy or low health literacy was a well-received and novel finding, highlighting the ability of mHealth apps to be better designed for diverse and underserved populations in research.

Cocreated features of the app, including the science-based sleep hacks and Sleep Stars gamified rewards system, were well-liked by participants, enabling them to better understand the science behind the recommendations and track their progress toward their goals. Participants may require the encouragement of authority figures to take part in Sleep Well, as they may not be aware of or seek out support for their sleep or mental health difficulties. The potential benefits and helpfulness of the app will need to be highlighted for any future implementation in order for participants to form positive expectations about Sleep Solved. Future work, adapting the Sleep Solved app to different age ranges or cultures, inclusion in adolescent health initiatives, or United Kingdom–wide scalable public health strategies may also extend the usefulness of the app, such as rollout across schools, colleges, universities, or inclusion in National Health Service digital health support programs.

## Supplementary material

10.2196/82410 Multimedia Appendix 1 Demographic characteristics of the nonrandomized feasibility trial sample (n=1048).

10.2196/82410 Multimedia Appendix 2 Quantified proportional summaries of themes, split by number of participants, number of codes, socioeconomic status, and ethnicity.

10.2196/82410 Checklist 1 COREQ checklist.

## Abbreviations

COREQ: Consolidated Criteria for Reporting Qualitative Research
LSOA: lower-layer super output area
mHealth: mobile health
PBA: person-based approach
SES: socioeconomic status
SHUTi: Sleep Healthy Using the Internet
SMILE: Smile for Life
SSI: single session intervention
YES: Youth Empowerment and Support
