# Supplementary material for: Inclusivity in Insomnia: Adolescents’ Perspectives on the Sleep Solved App: Qualitative Interview Study
Source: JMIR Form Res. 2026 Jun 10;10:e82410. doi: 10.2196/82410 (PMC13254758; doi:10.2196/82410)
Supplement: Multimedia Appendix 1 [file formative-v10-e82410-s001.docx]

**Demographic characteristics of the non-randomized feasibility trial sample (n=1048)**

**Table S1.** Demographic characteristics of the total non-randomized feasibility trial sample at baseline.

| Demographic characteristic | Total sample (n=1048) | |
| --- | --- | --- |
|  | *M* | *SD* |
| Age | 16.86 | 0.74 |
|  | *%* | *n* |
| Sex at birth |  |  |
| Female | 72.4 | 759 |
| Male | 27.6 | 289 |
| Gender |  |  |
| Female | 69.5 | 728 |
| Male | 27.3 | 286 |
| Prefer not to say | 2.8 | 29 |
| Ethnicity |  |  |
| Asian or Asian British | 8.6 | 90 |
| Bangladeshi | 12.2 | 11 |
| Chinese | 1.1 | 1 |
| Indian | 25.6 | 23 |
| Pakistani | 34.4 | 31 |
| Any other Asian background | 26.7 | 24 |
| Black, Black British, Caribbean or African | 4.5 | 47 |
| African | 72.3 | 34 |
| Caribbean | 14.9 | 7 |
| Any other Black, Black British or Caribbean background | 12.8 | 6 |
| Mixed or multiple ethnic groups | 3.5 | 37 |
| White and Asian | 29.7 | 11 |
| White and Black Caribbean | 27 | 10 |
| White and Black African | 2.7 | 1 |
| Any other Mixed or multiple ethnic background | 40.5 | 15 |
| White | 81.6 | 855 |
| English, Welsh, Scottish, Northern Irish or British | 92.4 | 790 |
| Gypsy or Irish Traveler | 0.2 | 2 |
| Irish | 0.8 | 7 |
| Roma | 0.4 | 3 |
| Any other White background | 6.2 | 53 |
| Other ethnic group | 1.8 | 19 |
| Arab | 31.6 | 6 |
| Any other ethnic group | 68.4 | 13 |
