# Supplementary material for: Inclusivity in Insomnia: Adolescents’ Perspectives on the Sleep Solved App: Qualitative Interview Study
Source: JMIR Form Res. 2026 Jun 10;10:e82410. doi: 10.2196/82410 (PMC13254758; doi:10.2196/82410)
Supplement: Multimedia Appendix 2 [file formative-v10-e82410-s002.docx]

**Quantified summary of qualitative themes**

**Table S2.** Quantified proportional summaries of themes, split by number of participants, number of codes, socioeconomic status and ethnicity.

| **Theme** | **Number of participants who mentioned this theme (n)** | **Number of codes within each theme** | **Socioeconomic status of participants** | **Self-reported ethnicity of participants** |
| --- | --- | --- | --- | --- |
| 1. Reasons for participating and expectations of the Sleep Solved app | 58 | 207 | n = 40 (68.9%) from the most deprived areas of the UK  n = 18 (31%) from the least deprived areas of the UK | n = 34 of White English, Welsh, Scottish, Northern Irish or British ethnicity  n = 15 of Asian or Asian British ethnicity  n = 3 of Black, Black British, Caribbean or Black African ethnicity  n = 2 “Other” ethnicity |
| 1. Experience of Sleep Solved | 47 | 602 | n = 32 (68%) from the most deprived areas of the UK  n = 15 (31%) from the least deprived areas of the UK | n = 30 of White English, Welsh, Scottish, Northern Irish or British ethnicity  n = 9 of Asian or Asian British ethnicity  n = 4 of mixed ethnicity  n = 2 of Black, Black British, Caribbean or Black African ethnicity  n = 2 of “Other” ethnicity |
| 1. Perceived benefits of Sleep Solved | 53 | 343 | n = 37 (69.8%) from the most deprived areas of the UK  n = 16 (30.1%) from the least deprived areas of the UK | n = 32 of White English, Welsh, Scottish, Northern Irish or British ethnicity  n = 13 of Asian or Asian British ethnicity  n = 4 of mixed ethnicity  n = 2 of Black, Black British, Caribbean or Black African ethnicity  n = 2 “Other” ethnicity |
